# From discovery to preparedness: reframing antimicrobial resistance as a global governance challenge

**DOI:** 10.3389/fpubh.2026.1965508

**Published:** 2026-10-01

**Authors:** Quan Khoi Nguyen, Jeffrey D. Mayne, Andrew W. Taylor-Robinson

**Affiliations:** 1 College of Health Sciences, VinUniversity, Hanoi, Vietnam; 2 Center for Innovations in Health Sciences, VinUniversity, Hanoi, Vietnam; 3 Johns Hopkins Bloomberg School of Public Health, Baltimore, MD, United States; 4 Department of Medicine, University of California, Los Angeles, Los Angeles, CA, United States; 5 Center for Global Health, Perelman School of Medicine, University of Pennsylvania, Philadelphia, PA, United States

**Keywords:** antibiotic, antimicrobial resistance (AMR), antimicrobial stewardship (AMS), global health governance, infection prevention and control (IPC), One Health framework

## Abstract

Almost a century after the discovery of penicillin, the antimicrobial resistance (AMR) crisis has become as much a failure of global governance as a biomedical challenge. Published Global Burden of Disease estimates for 2000–2021 show a persistent geographic hierarchy of mortality, a pattern consistent with AMR burden being embedded in health system capacity rather than due to pathogen evolution alone. Furthermore, the for-profit pharmaceutical model remains structurally misaligned with the clinical necessity of restricted antibiotic use. This Perspective argues that the next phase of AMR mitigation must pivot from a discovery-dominated paradigm to an integrated preparedness model in which innovation, stewardship, surveillance, and governance are collectively strengthened. We contend that success will be determined less by laboratory and technology breakthroughs than by the political commitment to decouple revenue from sales volume and to govern antimicrobial effectiveness as a shared, exhaustible resource. We propose a coordinated policy toolkit — integrating “push-pull” incentives and One Health surveillance — to protect the epidemiological utility of these finite resources. The antibiotics era began as a story of discovery, became a story of complacency, and must now become a story of stewardship and global governance.

## Introduction—Evolution of the antibiotics era

Nearly a century ago, the era of antibiotics began with a fortuitous observation. In 1928, the Scottish medical microbiologist Alexander Fleming noticed that a contaminating mold inhibited bacterial growth on a culture plate, leading to his discovery of penicillin (1). What followed was one of the most transformative periods in medical history: streptomycin for tuberculosis, tetracyclines for broad-spectrum infections, vancomycin for severe Gram-positive bacterial infections, and later cephalosporins and carbapenems that made modern surgery, intensive care, and chemotherapy possible (2). For a fleeting period of post-World War II industrial-scale production, it appeared that bacterial infections had been decisively conquered (3).

Yet, the victory proved short-lived. Antimicrobial resistance (AMR) has emerged to every class of antibiotic introduced into therapeutic use, and its clinical and environmental spread makes infections harder to treat (4). Notable examples include, in chronological order, penicillin-resistant staphylococci, methicillin-resistant *Staphylococcus aureus* (MRSA), and vancomycin-resistant enterococci (VRE) (5). This has turned what began as a triumph of discovery into a continuous arms race between human innovation and microbial adaptation under selective pressure (6). In this Perspective, we argue that the defining public health failure manifested by AMR is no longer primarily a shortage of scientific research and development, but the inability of health systems and global governance structures to manage antimicrobial effectiveness as a shared and exhaustible resource (7, 8). Discovery remains necessary; but our contention is that it is no longer sufficient.

### Geography of burden: structural persistence in AMR mortality

Estimates from Global Burden of Disease (GBD) data underscore both the scale and the persistent geography of AMR (9, 10). Comparison of estimates for 2000 and 2021 shows that although age-standardized AMR-related mortality has declined modestly, the ranking of burden has shifted only slightly (10). Mortality lessened somewhat everywhere, but the super-regions that were hit hardest at the turn of this century are still the most affected today. Sub-Saharan Africa remains the super-region with the highest death rates both associated with and attributable to resistant bloodstream infections — roughly two to three times those of high-income regions — while South Asia and parts of Southeast Asia continue to rank among the most affected regions. In contrast, the high-income regions plus Australasia and parts of East Asia consistently show the lowest mortality rates across both time points. To avoid ecological fallacy, these regional aggregates conceal substantial heterogeneity between and within countries in antibiotic access, surveillance capacity, governance, and agricultural systems, and should be read as a coarse map rather than a description of any individual setting.

This persistence of regional hierarchy over two decades is consistent with AMR-related mortality being shaped substantially by health system capacity (likely access to effective antibiotics, microbiology diagnostics, infection prevention and control (IPC), and critical care), rather than by pathogen evolution or pharmaceutical innovation cycles alone (11, 12). We infer from these patterns that the geography of AMR burden broadly mirrors the geography of health system strength and preparedness (13); a formal test of this association is beyond the scope of this Perspective but is a priority for future empirical work.

### Economic paradox: why the market discourages antibiotic research and development

While the slowdown in antibiotic discovery is a scientific problem, it is equally an economic one (14, 15). From a for-profit standpoint, antibiotics make for unattractive products: they are prescribed in short courses, reserved for severe infections, and inevitably lose effectiveness as resistance develops. Furthermore, antimicrobial stewardship (AMS) programs also limit revenue through appropriately restricted antibiotic use. As a result, expected returns are modest compared with drugs for chronic conditions. Estimates of net present value for a newly approved antibiotic range from roughly –US$50 million to +US$100 million, compared with US$700 million to over US$1 billion for a typical oncologic agent, or US$1.15 billion for a musculoskeletal product (14–17). These estimates come from different years and modeling assumptions and are sensitive to development cost, market size and time horizon; therefore, they indicate direction rather than precise magnitude (15, 16).

Over the past two decades, many major firms have scaled back or abandoned antibiotic pipelines (15). Several companies developing novel antibiotics have entered bankruptcy soon after regulatory approval, highlighting the absence of sustainable post-market revenue models (18). New agents must compete with inexpensive generics, while resistance emerges to nearly every class over time, shortening commercial lifespans and reinforcing industry caution. The upshot is a persistent paradox: society urgently needs new antibiotics, yet the market discourages their development (Supplementary Figure 1, left panel).

### Beyond discovery: re-envisioning AMR as a systems-level governance problem

The contemporary AMR crisis is, in our assessment, as much a failure of global governance as a shortfall in biomedical discovery. While the first century of antibiotics was defined by the search for new molecules, the next must be defined by preserving antibiotic effectiveness once new drugs reach the market (14, 19). Table 1 contrasts these two paradigms across their core premises, economic models, regulatory focus, and success metrics; the contrast is heuristic, and the preparedness paradigm incorporates rather than replaces discovery. Faced with the “silent pandemic” of AMR, it is critical for the global health community to prioritize preparedness — a multifaceted approach encompassing robust surveillance capacity, IPC programs, laboratory infrastructure, regulatory coordination, and sustainable financing. Currently, global interventions remain misaligned with the scale of the threat: a governance analysis of 114 countries revealed substantial gaps in national action plans, especially regarding accountability mechanisms, monitoring and evaluation (19). Without structural reform, the worldwide response remains uneven and ineffective.

**Table 1:** Two paradigms of the antibiotics era: from discovery to preparedness.

Dimension	Discovery paradigm (20^th^ century)	Preparedness/governance paradigm (21^st^ century)
Core premise	Scarcity of *molecules* — resistance is an R&D problem solved by the next drug	Scarcity of effectiveness — resistance is a governance problem of a shared, exhaustible resource
Economic model	For-profit, volume-based sales; revenue depending on units sold	Decoupled/subscription (“Netflix-style”); revenue delinked from volume; tiered global financing
Primary goal	Bring new agents to market	Preserve the epidemiological utility of existing + new agents
Resource logic	Antibiotics as market commodity	Antibiotics effectiveness as common-pool resource, antibiotics governed as public utility (27)
Regulatory focus	Approval, patents, market exclusivity	Stewardship (AMS), IPC, cross-sectoral One Health enforcement, treaty-based accountability
Key actors	Pharmaceutical industry, national regulators	Multilateral bodies, national health systems, human–animal–environment sectors
Geography of effort	High-income R&D hubs	LMIC health system capacity, WASH, decentralized surveillance
Success metric	Number of new drugs approved; sales	Burden reduction + resistance rates held stable; strength of surveillance/AMS/IPC architecture
Failure mode	Empty pipeline; developer insolvency	Free-rider collapse; efficacy “harvested” faster than replenished
Time horizon	Product lifecycle (patent term)	Intergenerational (efficacy preserved for future patients)

AMS, antimicrobial stewardship; IPC, infection prevention and control; LMIC, low- and middle-income country; R&D, research and development; WASH, water, sanitation and hygiene. Please note that the contrast is heuristic: the preparedness paradigm incorporates, rather than replaces, continued antibiotic discovery.

Central to this challenge is the disconnect between public health needs and economic incentives (20). The preservation of antimicrobial effectiveness is a global public good, yet current reimbursement models often depend on sales volume, creating a paradox where commercial success is inversely related to the clinical necessity of restricted use. In this context, the introduction of novel agents alone cannot resolve the crisis (21). Crucially, while high-income countries may have the fiscal capacity to pilot pioneering “pull” incentives, including a ‘Netflix-style' subscription model, this creates a potential for ‘free rider' risk, where some nations benefit from the existence of a revitalized pipeline without contributing to its maintenance (8, 19). The governance challenge here lies in designing a tiered contribution framework that ensures middle-income countries participate in sustaining these global funds without compromising their domestic health equity or access to the very drugs they are helping to subsidize (19, 22). AMR must therefore be re-envisioned as a systems-level governance problem requiring a coordinated global policy that protects the epidemiological utility of these finite resources. Figure 1 summarizes, as a conceptual synthesis, this shift, the structural drivers behind it, and the coordinated policy response for which it calls.

**Figure 1:**
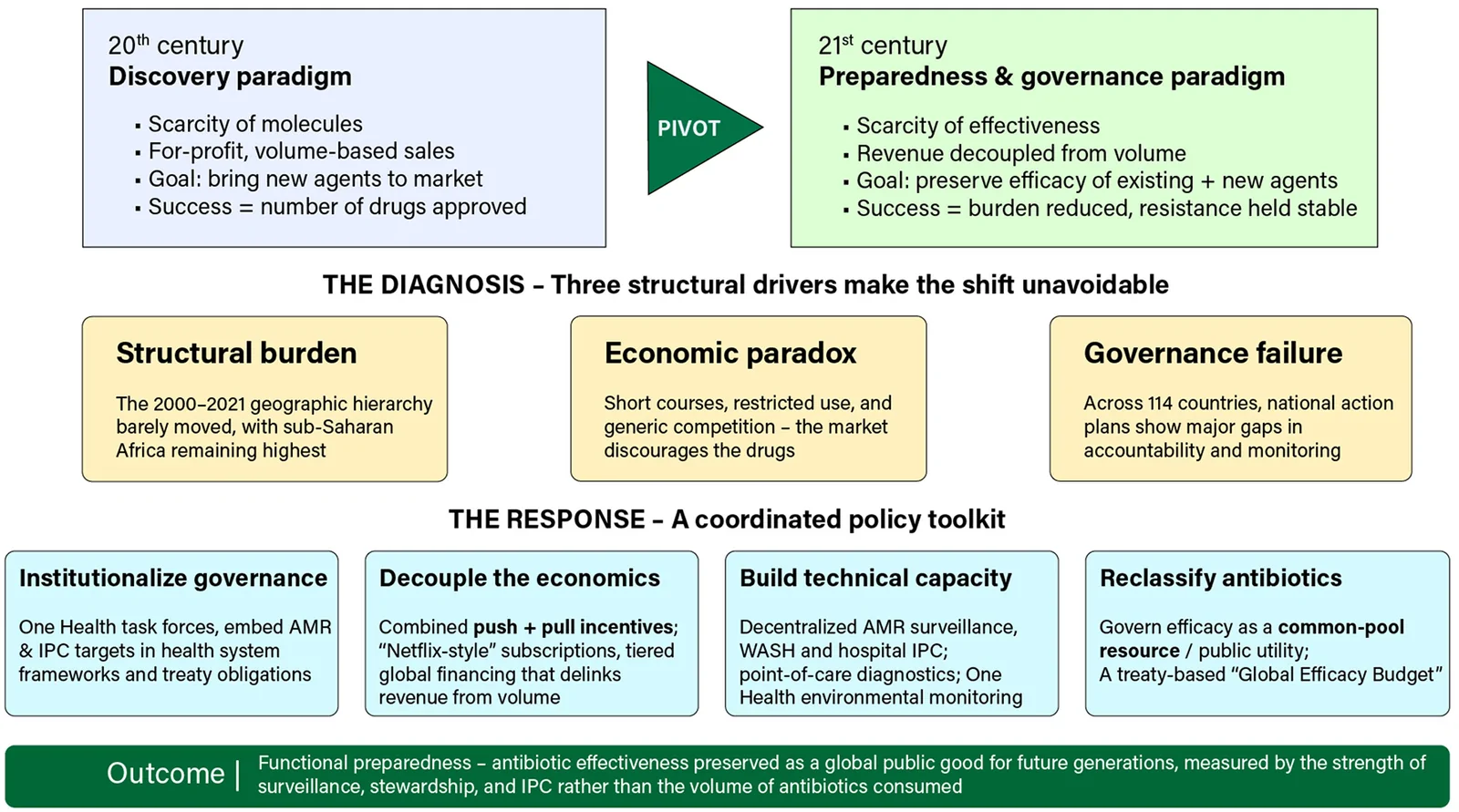
A conceptual framework from discovery to preparedness: reframing antimicrobial resistance (AMR) as a global governance challenge. **The upper panel** contrasts the 20^th^ century discovery paradigm — in which resistance is a research-and-development problem solved by the next molecule — with a 21^st^ century preparedness-and-governance paradigm, in which the scarce resource is antibiotic effectiveness and revenue is decoupled from sales volume. Three structural drivers make the shift unavoidable: the geographic burden of AMR tracks health-system capacity rather than pathogen evolution; the market discourages development of the drugs society most needs; and the global governance response remains fragmented across countries and sectors. **The lower panel** outlines a coordinated policy toolkit — institutionalizing governance and accountability, decoupling the economics of antibiotic supply, strengthening technical and surveillance capacity, and reclassifying antibiotics as a common-pool resource — directed toward functional preparedness, in which antibiotic effectiveness is preserved as a global public good. AMR, antimicrobial resistance; IPC, infection prevention and control; WASH, water, sanitation and hygiene.

### Policy toolkit: aligning incentives through structural reform

A substantial policy toolkit already exists to address this structural mismatch, yet implementation remains fragmented. Antibiotic innovation policies converge on a combined package of “push” and “pull” incentives: push mechanisms such as public research funding, grants, tax credits, public–private partnerships, shared compound libraries, and open-science platforms to lower development costs; and pull mechanisms that reward successful products through market-entry rewards, subscription-style payments, advance market commitments, patent buyouts, transferable exclusivity extensions, and regulatory acceleration (14–16). Hybrid models that unlink revenue from sales — now piloted in the UK and Sweden and proposed elsewhere — are increasingly viewed as essential to reconcile AMS with commercial sustainability, effectively transitioning antibiotics from products of consumption to assets of preparedness (15, 16, 22). Their affordability for low- and middle-income countries (LMICs) and their effect on the pipeline remain to be demonstrated, and no single pull mechanism will suit every setting (16, 22). Governance reviews emphasize the need for stronger accountability mechanisms, sustainable domestic financing of national action plans, and clearer global coordination across clinical, veterinary, and environmental sectors (i.e., a One Health framework) (19).

### Governing antibiotics as a critical public utility

New discovery approaches such as deep learning networks may expand the antibiotic pipeline (23–25), but without parallel advances in AMS, surveillance, and financing models, even novel agents will lose effectiveness (6, 8). Immediate next steps include scaling subscription-based payment pilots, establishing multinational financing mechanisms for market-entry rewards, integrating AMR surveillance across hospital and laboratory networks, and embedding AMS and IPC metrics into health system performance frameworks (26–28). Antibiotics as products are ordinary private goods; what is shared is antimicrobial effectiveness (i.e., the population-level susceptibility of pathogens to available drugs). This is subtractable and difficult to exclude others from, the two defining features of a common-pool resource (26, 27). Its preservation is a global public good, and “public utility” denotes the regulatory model we propose for governing it. Like ocean fisheries or climate stability, it is depleted by uncoordinated exploitation for immediate gain — a classic collective-action failure (27). We therefore propose, as a conceptual framework rather than an operational scheme, a ‘Global Efficacy Budget': a negotiated, time-bound allowance of antimicrobial consumption — expressed, for example, in defined daily doses per 1,000 inhabitants per day for humans and milligrams per population correction unit for animals, weighted toward WHO Watch and Reserve agents (29) — allocated nationally, with explicit access floors for countries whose use falls below clinical need. Its logic parallels a carbon budget, and the Paris Agreement's lessons on differentiated responsibility, transparency and periodic ratcheting apply (30). Metrics, allocation rules, enforcement and veterinary integration remain to be developed; our claim is only that treaty-based oversight and collective investment of the kind afforded to other exhaustible health infrastructure (7) are necessary, because a framework without consequences for non-compliance is likely to stall in the same way as have voluntary national action plans (19).

Some would argue that scientific stagnation remains the principal bottleneck to effective infection control, yet to date resistance has emerged to every antibiotic class (6), so the absence of governance reform would undermine even a revitalized pipeline. In our considered opinion, therefore, the next phase of AMR mitigation will be determined less by laboratory breakthroughs alone than by political commitment to govern antimicrobial effectiveness as a vulnerable public utility rather than a standard market commodity.

### Policy recommendations for structural AMR mitigation

To make these recommendations actionable, the following framework outlines a transition from a discovery-dominated model toward proactive governance of antibiotics as an exhaustible clinical resource, alongside continued innovation; principal actors and an indicative time horizon are given for each.

### 1 . Institutionalizing governance and accountability (national governments, WHO/Quadripartite; immediate–medium term)

Global and national responses must move beyond voluntary participation toward formalized accountability mechanisms. National governments should integrate specific AMR targets and IPC indices directly into their broader health system performance frameworks (19, 28). The 2024 United Nations General Assembly political declaration, which set a 10% reduction in AMR-associated deaths by 2030, a minimum 70% of human antibiotic use from the WHO Access group, and funded national action plans in at least 60% of countries (31), provides measurable targets to which such frameworks can be tied. This includes establishing permanent, inter-ministerial task forces that synchronize regulatory efforts across human, animal, and environmental health sectors — a true One Health integration. By embedding these metrics into national health budgets and international treaty obligations (7), the global community can ensure that AMR preparedness is treated with the same fiscal and political priority as pandemic prevention and climate change mitigation.

### 2 . Implementing economic reform and market decoupling (national payers and finance ministries, G7/G20, industry; medium term)

The fundamental mismatch between public health needs and the for-profit pharmaceutical model requires a substantial restructuring of how antibiotics are reimbursed. Policymakers should prioritize the scaling of ‘pull' incentives, such as subscription-style payment models, which provide manufacturers with a fixed annual fee for access to a drug regardless of the volume sold (22). This “Netflix-style” approach effectively decouples revenue from sales, removing the commercial pressure to over-market broad-spectrum agents. Furthermore, establishing multinational financing mechanisms for market-entry rewards can provide the necessary “pull” for private pharmaceutical companies (8), ensuring that novel agents targeting World Health Organization priority pathogens are both developed and preserved for restricted, appropriate use.

### 3 . Strengthening technical infrastructure and surveillance (ministries of health and agriculture, funders; immediate and continuing)

Reliable governance is impossible without granular, actionable data. Governments must invest in decentralized, laboratory-based surveillance networks, particularly in high-burden settings in LMICs, in order to move from passive facility-based reporting to active epidemiological mapping (11, 32). This technical expansion must be paired with the subsidized deployment of point-of-care diagnostics to reduce empiric prescribing (e.g., in Vietnamese primary care, point-of-care C-reactive protein testing reduced antibiotic prescribing for non-severe acute respiratory infections in both an individually randomized trial and a pragmatic cluster-randomized implementation trial, although uptake in the latter was low) (33, 34). Additionally, the policy focus must return to the structural basics of public health: prioritizing universal access to clean water, sanitation, and rigorous hospital IPC as primary and cost-effective barriers against the transmission of drug-resistant pathogens. Modeling suggests that improved healthcare IPC and universal water, sanitation and hygiene could prevent an estimated 337,000 and 248,000 AMR-associated deaths a year, respectively, in LMICs (35); the 2024 political declaration sets 2030 targets for both (31). Stewardship in these settings is two-sided: inappropriate use and lack of access to effective antibiotics coexist, with the latter remaining a major cause of avertable deaths (11, 36). Any consumption budget must therefore set access floors as well as ceilings, so that stewardship in high-consumption settings is not financed by rationing in under-served ones.

Furthermore, a systems-level approach must extend beyond the clinic to address the governance of antimicrobial use in commercial agriculture and aquaculture: animals raised for food are estimated to account for roughly 73% of all antimicrobials used worldwide (37, 38). Technical infrastructure for AMR cannot be considered complete without integrated “One Health” surveillance that links veterinary prescription data and environmental runoff monitoring with human clinical isolates (39). The current governance failure is exemplified by the regulatory “patchwork” where life-saving human antibiotics are still utilized as growth promoters for livestock in several jurisdictions. Strengthening infrastructure thus requires not only laboratory capacity but also the regulatory “teeth” to enforce cross-sectoral standards; this ensures that the environmental and animal-health drivers of resistance are no longer externalized by the food production industry at the expense of global health security (13, 39).

### 4 . Regulating antibiotics as critical infrastructure (UN member states, WHO; long term)

Ultimately, the legal and economic status of antimicrobials must be reclassified. Instead of treating antibiotics as ordinary market commodities, their effectiveness should be governed as exhaustible health infrastructure whose preservation is a global public good (7, 40). This shift justifies sustained public-sector investment in the entire “lifecycle” of an antibiotic — from early-stage discovery grants (“push” incentives) to post-market AMS and environmental monitoring. By regulating these finite resources with the same oversight afforded to water systems or power grids, the global health community can protect their long-term epidemiological utility for future generations (8, 40) (Supplementary Figure 1, right panel).

## Discussion

The persistence of regional AMR-related mortality hierarchies over the past two decades suggests that resistance is not merely a biological phenomenon but also a marker of broader structural inequality (41). In many high-burden settings across sub-Saharan Africa and South and Southeast Asia (noting wide variation between and within countries), the challenge is defined by a triple threat: limited access to often more powerful second-line therapies (11), insufficient diagnostic laboratory infrastructure for guided prescribing (thereby forcing clinicians to rely on empiric therapy) (42), and the pervasive environmental transmission of resistant pathogens through inadequate sanitation (43). Consequently, promoting new molecules into these “leaky” systems without addressing the underlying One Health determinants — such as water quality and animal health oversight (39) — will likely result in the rapid obsolescence of even the most advanced antibiotics.

Moreover, failure to decouple antibiotic revenue from sales volume represents a fundamental misalignment of global health priorities (15). While “push” and “pull” incentives are often discussed as economic tools, they are in fact essential governance instruments (8). The next century of bacterial IPC must therefore be defined by “functional preparedness” — a state in which the success of a health system is measured not by the volume of antibiotics consumed, but by the strength of the surveillance, AMS, and IPC architectures that preserve them (44). As AMR respects no borders, preserving antibiotic susceptibility should be reframed from a national priority into a global public good (7). Legal scholarship has argued that this reframing provides a normative basis for a binding international instrument on AMR with accountability mechanisms of the kind used in other global health and environmental agreements (30, 45); how such an instrument would interact with intellectual property and trade rules is a matter for negotiation rather than assertion. Our narrower claim is that the exhaustible nature of antimicrobials justifies placing their stewardship within an international mandate, protected from the commercial imperatives of individual firms and from uncoordinated national regulation (8, 19).

## Conclusion

While a century of innovation built a vast antimicrobial arsenal, our collective failure to safeguard these drugs suggests that the binding constraint on public health today is governance at least as much as it is pharmaceutical discovery. The immediate challenge is not only bringing novel agents to market, but institutionalizing current pilot frameworks (subscription-based reimbursement, tiered multinational financing of market-entry rewards, and cross-sector One Health data sharing) into binding global accountability before AMR renders existing therapies obsolete. Scaling these models would secure antimicrobial effectiveness as a protected shared asset; failing to do so risks a pipeline becoming exhausted as quickly as it is replenished, returning global health to a pre-antibiotic state characterized by infections that we cannot treat.

## Data Availability

Publicly available datasets were analyzed in this study. This data can be found at: https://vizhub.healthdata.org/microbe/.

## Supplementary material

Supplementary Figure 1 Governance blueprint for antimicrobial stewardship in the post-discovery era. **Left** (discovery paradigm/complacency era): historically, AMR mitigation has focused linearly on laboratory breakthroughs and market-driven drug discovery. Analysis of Global Burden of Disease (GBD) data (2000–2021) demonstrates a persistent geographic hierarchy in AMR mortality, illustrating that disease burden is structurally embedded in regional health system capacity rather than pathogen genetics alone. The traditional for-profit pharmaceutical sales model — which relies on sales volume for revenue — remains fundamentally incompatible with the clinical necessity of strict antibiotic stewardship and restricted consumption. **Center** (conceptual shift): the necessary paradigm pivot from viewing AMR strictly as an evolving biomedical/microbiological threat to managing it as a structural failure of global governance. **Right** (preparedness model/governance era): proposed governance architecture anchored by a coordinated policy toolkit comprising sustained political commitment, integrated One Health surveillance (linking human, veterinary, and environmental monitoring), and targeted “push-pull” financial incentives. Decoupling pharmaceutical revenues from sales volume enables antimicrobials to be regulated and preserved as a vulnerable global public utility, driving equitable reductions in global AMR mortality.
